# Determining Field Insecticide Efficacy on Whiteflies with Maximum Dose Bioassays

**DOI:** 10.3390/insects14060510

**Published:** 2023-06-01

**Authors:** Paulo S. G. Cremonez, Jermaine D. Perier, Alvin M. Simmons, David G. Riley

**Affiliations:** 1 Department of Entomology, University of Georgia Tifton Campus, Tifton, GA 31793, USA; jermaine.perier@uga.edu (J.D.P.); dgr@uga.edu (D.G.R.); 2 U.S. Vegetable Laboratory, Agricultural Research Service, United States Department of Agriculture, 2700 Savannah Highway, Charleston, SC 29414, USA; alvin.simmons@usda.gov

**Keywords:** insecticide resistance management (IRM), maximum-dose bioassay, sweetpotato whitefly, *Bemisia tabaci*

## Abstract

**Simple Summary:**

The sweet potato whitefly is a major pest of crops worldwide, causing significant damage. To control this pest, farmers often use insecticides, but the effectiveness of these treatments can vary depending on resistant population dynamics. We investigated the efficacy of insecticides on adult whiteflies in the field and laboratory conditions using maximum labeled rate methods. Fast-acting insecticides were more effective in controlling whitefly adults, and bioassays could be an effective tool for determining efficacy prior to expensive field applications. These findings will be valuable to farmers and researchers seeking to optimize control strategies for whiteflies, helping to reduce crop damage and improve yields.

**Abstract:**

We conducted a rapid bioassay method to assess insecticide efficacy for controlling adult sweetpotato whitefly *Bemisia tabaci* in squash and cucumber crops before insecticide applications. The study aimed to evaluate the accuracy of a 24-hour laboratory bioassay in determining maximum dose insecticide efficacy in the field. Ten insecticides were evaluated using leaf-dip bioassays, and their effectiveness was tested across eight cucurbit field experiments in Georgia, USA, during the 2021 and 2022 field seasons. The maximum dose, defined as the highest labeled rate of an insecticide diluted in the equivalent of 935 L ha^−1^ of water, was used for all bioassays. Adult survival observed in the bioassay was compared to adult field count-based survival 24 h after treatment. A low concentration (1/10 rate) was used for imidacloprid, flupyradifurone, pyriproxyfen, and cyantraniliprole to assess insecticide tolerance in the whitefly population. Overall, significant positive correlation between laboratory bioassay and field efficacy was reported, explaining 50–91% of the observed variation. The addition of the low dosage was helpful, indicating that no rate response was consistent with susceptibility to the tested insecticide, while a rate response was associated with a loss of susceptibility between 2021 and 2022.

## 1. Introduction

The sweetpotato whitefly, *Bemisia tabaci* (Gennadius) (Hemiptera: Aleyrodidae), is an important pest of several agricultural and vegetable crops worldwide [1,2]. Whiteflies have mouthparts adapted for piercing–sucking the sap from the phloem of leaves and are responsible for various types of damage, including direct feeding injuries such as reduced yields and irregular fruit ripening, the transmission of plant viruses, and the excretion of sugary honeydew which facilitates sooty mold growth [3]. These types of damage often result in significant economic strain on producers due to the high cost of whitefly insecticide control and equally high losses in production value. Notably, the piercing–sucking feeding nature of *B. tabaci* often limits effective insecticides to those with systemic activity in the plant, as contact application methods can be less effective for some life stages [4].

Certain members of the *Bemisia tabaci* species complex are known to be efficient in developing resistance to synthetic insecticides of many chemical classes [2]. The Middle East–Asia Minor 1 population (=Biotype B or *Bemisia argentifolii* Bellows & Perring) is primarily controlled with regular insecticide applications in Florida and the neighboring states [5]. Resistance to commonly used insecticide products, such as imidacloprid and thiamethoxam, has been a historical issue in regional populations [5,6]. Therefore, strategies to reduce the loss of practical insecticide efficiency, such as insecticide resistance management (IRM) tactics, are increasingly needed [7]. Toxicological laboratory bioassays aim to measure an insecticide’s activity on insect survival using standardized methods. Confounding factors that are naturally present in the field (e.g., climatic variations, insect dispersal, and relationships of natural enemies) are, with a bioassay, nullified or minimized, allowing for a direct focus on the insecticide response. This study aimed to test the general hypothesis that conducting laboratory bioassays on whitefly populations before insecticide application can accurately determine product efficacy in the field. Assessing whitefly insecticide toxicity is a potentially practical tool for aiding growers in making insecticide control decisions [4,7,8,9,10,11,12].

Combining quick, easy-to-use bioassays with effective chemical rotation options could be advantageous for IRM’s overall efficiency. Our main hypothesis tested was that the efficacy of selected insecticides in the maximum dose bioassay would correlate with the survival estimate of adults who occurred in the field after treatment. A sub-objective of this hypothesis for a subset of insecticides was that adding a low concentration to the bioassay would improve the interpretation of laboratory and field efficacy measurements, with a significant rate response suggesting a loss in field efficacy.

## 2. Materials and Methods

### 2.1. Field Conditions

Four standard insecticide efficacy field trials were conducted per year, two in squash, *Cucurbita pepo* (L), and two in cucumber, *Cucumis sativus* L., at the University of Georgia Coastal Plain Experiment Station in Tifton, GA (31°30′53″ N, 83°32′51″ W farm site) during the summers of 2021 and 2022. The test plants were seeded from late June to early August each year to coincide with annual increases in whitefly populations in southern Georgia [13]. The field spray trials were performed in tandem with laboratory bioassays evaluating whitefly response to the same field-tested insecticides at a proportional mixture concentration (i.e., converted from mL or g of active ingredient [a.i.] hectare^−1^ to mL or g a.i. L^−1^). These laboratory bioassays on populations of whiteflies collected from the same test field plots were conducted using clean cotton seedlings grown in insect-free growth chambers (30 ± 2 °C, 50 ± 5% RH, 14 L: 10 D h photoperiod) as the host plant test media. We compared cotton leaf bioassay results with standard counting in various cucurbit treated field plots to see if laboratory whitefly survival correlated with field population numbers. The tested insecticides used in field tests and bioassay treatments are summarized in Table 1. A total of eight field experiments were conducted; four were in squash (“Yellow Crookneck”), and four were in cucumber (“Straight 8”). All crops were seeded at 30 cm spacing and were fertilized pre-planting with 560 kg 10-10-10 (NPK) ha^−1^ incorporated into Tift Pebbly Clay Loam soil type with overhead irrigation as needed. Pre-spray bioassays were conducted on the crop after the five-expanded-terminal true-leaf growth stage to keep the crop stage consistent between experiments.

### 2.2. Laboratory Bioassays

A cotton seedling tube system method was used, as described by Sparks et al. [7]. Cotton seedlings with at least one mature true leaf were carefully removed from the soil medium. Any other leaves were detached, and only one remaining leaf was dipped in the insecticide mixture or pure distilled water for the control (check). After air drying, the roots of each plant were enclosed in a scintillation vial (V = 20 mL) containing tap water and 0.5% 24-8-16 fertilizer (Miracle-Gro^®^ Plant Food, Marysville, OH, USA), enclosed with cotton wool and sealed with Parafilm^®^. The leaf was exposed to keep the plant alive during the evaluation period. For the bioassays, we used proportional treatment concentrations of the per hectare rate (Table 1) in the equivalent of 935 L ha^−1^ water spray volume as a maximum labeled rate, herein referred to as the “high” (H) concentration. An additional “low” (L) concentration, defined as one-tenth of the high concentration, was also used for Admire (imidacloprid), Sivanto Prime (flupyradifurone), Knack (pyriproxyfen), and Exirel (cyantraniliprole) to evaluate if an additional concentration would improve sensitivity in the insecticide response measurement.

**Figure 1:**
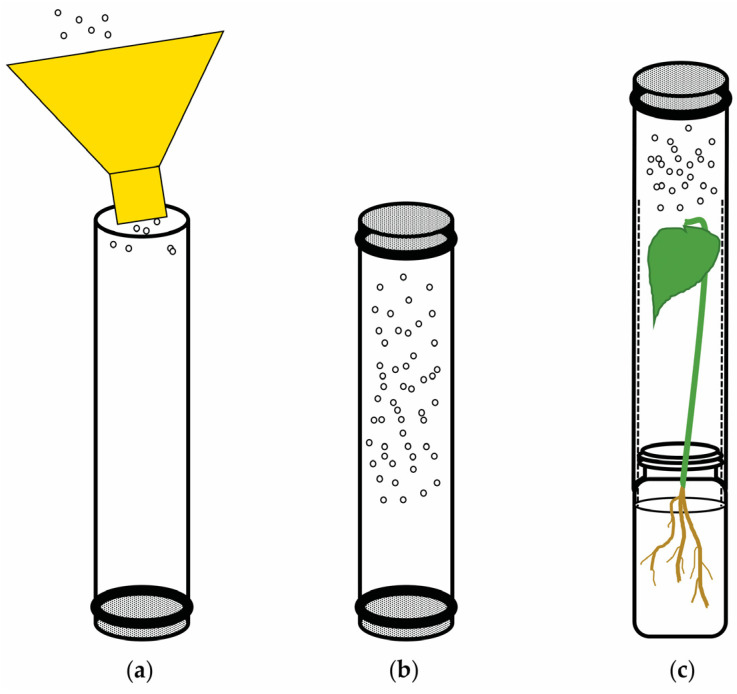
Schematic representation of *Bemisia tabaci* adult collection and cotton seedling-tube system bioassay method adapted from Sparks et al. [7]. (**a**) whiteflies are collected from the field with a funnel-tube apparatus; (**b**) tube with whiteflies is sealed in both ends with nylon chiffon fabric for transportation to laboratory; (**c**) a pre-treated cotton seedling is held by an open clear tube sleeve (dashed lines) to facilitate introduction of the tube with whiteflies with free access to the treated leaf.

Each adult bioassay was performed concomitantly with its relative field trial. Whiteflies used in the bioassays were collected from the relative cucurbit field plots before applying insecticides. A sample of approximately 50 unsexed *B. tabaci* adults of unknown ages was collected from the entire field in ClearTec^®^ tubes (V = 130 mL, ClearTec Packaging, Park Hills, MO, USA) with holes covered with nylon chiffon fabric for ventilation and was immediately carried back to the laboratory. The choice of this sample size was based on the need to account for the potential effects of a mixed-age population while ensuring that a substantial number of active whiteflies per experimental unit from the field were tested in the laboratory. The tubes containing field-collected whitefly adults were affixed to the previously treated cotton seedling tube systems with a tube sleeve allowing enclosed adults free access to the abaxial leaf surface (Figure 1). Each tube served as an experimental unit, with six repetitions per treatment per bioassay. The tubes were maintained in controlled conditions (25 ± 2 °C, 60 ± 5% RH, 24 L: 0 D h photoperiod). Adult survival was assessed at 24 h after initial treatment and control exposure. A pre-sampling was conducted a day before the application to confirm a statistically even distribution of whiteflies throughout the plots.

### 2.3. Field Trials

A field insecticide spray was carried out simultaneously with the 24 h laboratory bioassay reading; five plants were selected randomly per plot and were inspected 24 h after application, during which the numbers of live whitefly adults were recorded from a single leaf per plant using the standard leaf-turn technique of the third specific leaf node from the apical meristem [14]. Field treatments were applied with a tractor-mounted, pressurized air sprayer at 413.7 kPa (60 psi) with three TX-18 hollow cone tips (Spraying Systems Company, Bessemer, AL, USA) per row and a spray volume of 496 L ha^−1^. The treated plot size was two rows measuring 18.3 m long × 1.8 m wide, with plant spacing of 0.3 m, totaling approximately 120 plants per plot. This was replicated four times for each crop, over two summer seasons, in a randomized complete block design.

### 2.4. Statistical Analysis

All trials and bioassays data were analyzed in SAS^®^ Enterprise Guide v. 8.3 (SAS Institute Inc., Cary, NC, USA) using PROC GLM for generalized linear model analysis complemented with Tukey’s test for mean comparison (*p* < 0.05). Additionally, the PROC CORR procedure was used for Pearson correlation coefficient (ρ) analysis, and PROC REG procedure for linear regression analysis (variables laboratory proportional survival [%] and field estimate proportional survival [%, calculated as the ratio between the observed number by the maximum number in the test multiplied by 100]). In addition, we compared adult survival in the bioassay to adult counts in the field as an estimate for insecticide efficacy because an accurate count of dead adults in the field treatments could not be reliably made. The results of both squash and cucumber experiments were similar within the crop system. Therefore, the combined average variables were used for a single correlation analysis (n = 12) for each crop. Finally, a comparison (Tukey’s test, *p* < 0.05) was also made of the high and low concentrations of the subset of insecticides previously listed to the adult survivorship in the field (a percentage of the high adult count in the untreated plots).

## 3. Results

In the field pre-sample collection (n = 55 for each test), there was no significant pre-treatment effect either in 2021 (range count = 2–16, F_13, 206_ = 1.27, *p* = 0.235) or in 2022 (range count = 39–128, F_13, 206_ = 0.77, *p* = 0.693). For the pre-treatment collected whitefly bioassay and post-treatment assessed field survival estimates, a summary of the general replication effects followed by data analysis is presented (Table 2).

In most cases, the insecticide treatments had significantly higher whitefly adult mortality than the control in both seasons on squash (Table 3) and cucumber (Table 4). Consistently, the insecticides dinotefuran (4A), flupyradifurone (4D), and cyantraniliprole (28) were more efficacious against the adults, with survival values lower than 15% at 24 h after exposure. The same insecticides proved to be more efficient in the field 24 h post-spray treatment in all cases, except for dinotefuran and flupyradifurone in cucumber 3 (2022) and cyantraniliprole in cucumber 4 (2022).

**Figure 2:**
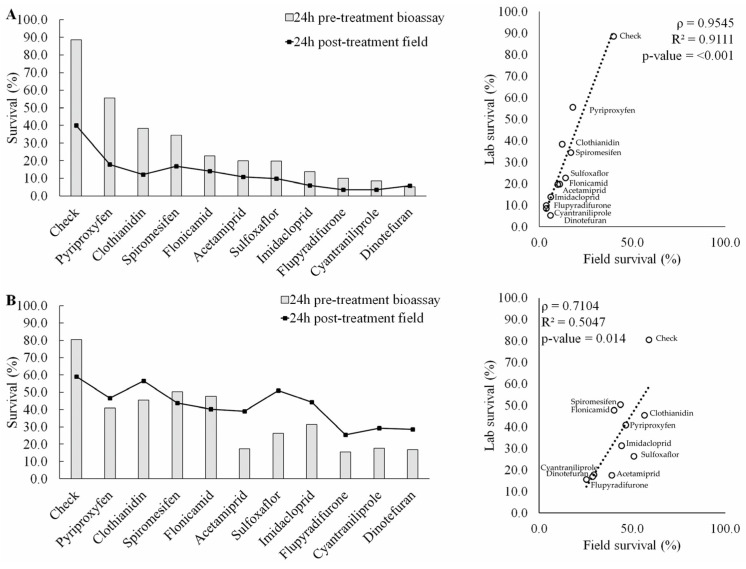
Correlation and regression analysis of insecticide efficacy in squash for adult *Bemisia tabaci* between laboratory bioassays and squash field spray experiments. (**A**) 2021 season; (**B**) 2022 season. ρ = Pearson correlation coefficient; R^2^ = regression coefficient.

**Figure 3:**
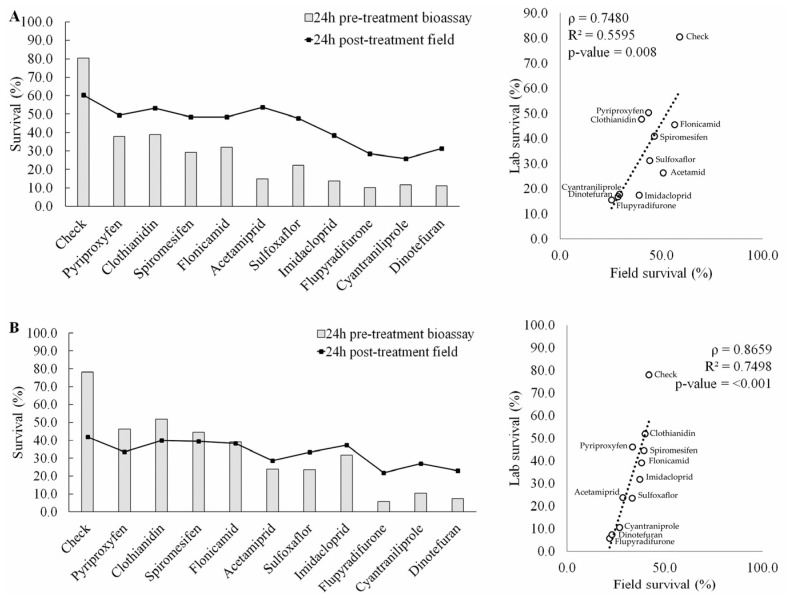
Correlation and regression analysis of insecticide efficacy in cucumber for adult *Bemisia tabaci* between laboratory bioassays and cucumber field spray experiments. (**A**) 2021 season; (**B**) 2022 season. ρ = Pearson correlation coefficient; R^2^ = regression coefficient.

Positive correlations were observed across all treatments between the pre-treatment laboratory bioassay survival and the post-field spray survival estimate analysis in both squash and cucumber in 2021 (ρ = 0.95 and 0.71, respectively) and 2022 (ρ = 0.75 and 0.87) (Figure 2 and Figure 3). The regression analysis used to compare the laboratory and field results of adult whitefly survival provided a significant relationship (from ρ = 0.71, R^2^ = 0.50, *p* = 0.014 [cucumber 2021] to ρ = 0.95, R^2^ = 0.91, *p* < 0.001 [squash 2021]) between the whitefly adult survival and field counts based on treatment distribution. Efficacy analysis based on bioassay and proportional field survival resulted in very similar ranks of insecticide averaged over both crops and both years. Except for squash from 2021 (Figure 2A), we observed better laboratory survival in the untreated check (Figure 2B and Figure 3A,B). This bias, however, did not significantly disrupt the correlation in any of the crop systems or seasons analyzed. The untreated check results were consistently high enough to standardize the regression analysis. At the same time, insecticides that were generally efficient against whiteflies, such as dinotefuran, cyantraniliprole, and flupyradifurone, resulted in consistently low laboratory and field survival, thus contributing to a consistently low value for the regression analysis of the ranked efficacy. Low and moderately efficacious insecticides tended to settle in the median portion of the ranked regression, but there was more variability in this group.

**Figure 4:**
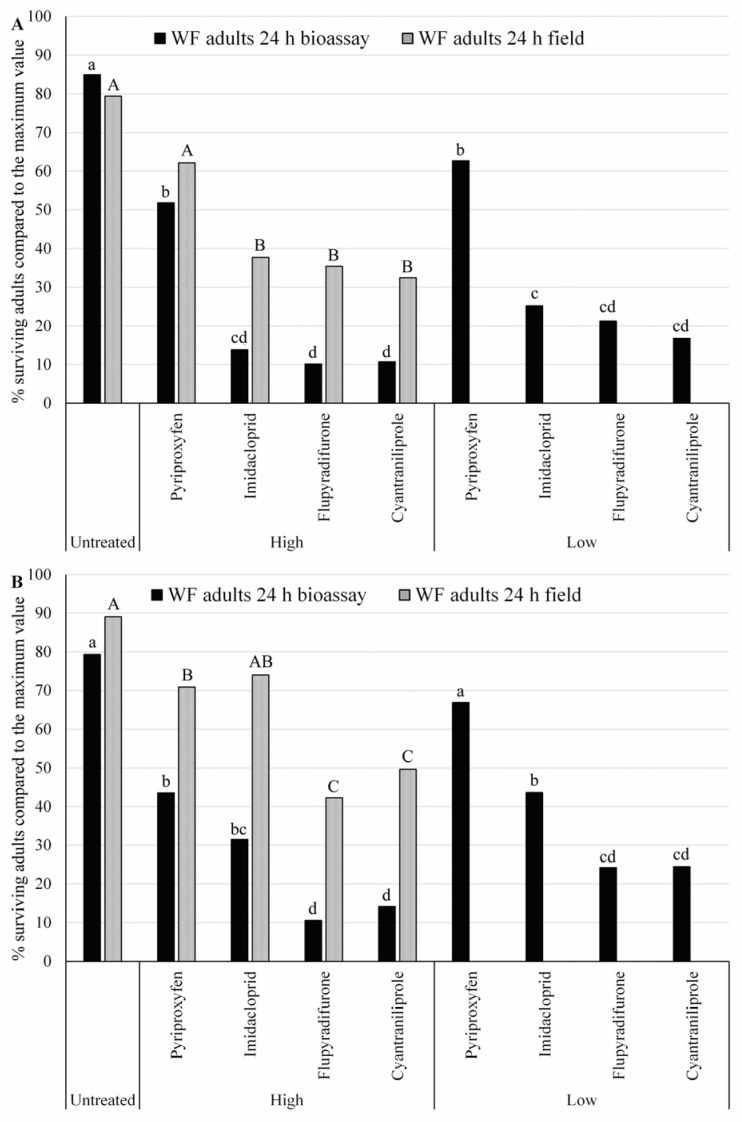
Comparison of insecticide efficacy for adult *Bemisia tabaci* between high–low laboratory bioassays and the average over four field spray experiments. (**A**) 2021 field season; (**B**) 2022 field season; bars with different uppercase or lowercase letters are significantly different (*p* < 0.05, Tukey’s test).

We added a low concentration to the maximum dose bioassay in the pre-treatment laboratory bioassay, then compared it with field numbers that provided more resolution in terms of potential whitefly response (Figure 4). In general, 2021 field results based on the high concentrations (max label rate) were consistently paired with the analogous bioassay survival in both high and low concentration results (Figure 4A). Based on the bioassay results, significantly higher efficacy of the imidacloprid, cyantraniliprole, and flupyradifurone compared to pyriproxyfen was observed in 2021. However, in 2022, field results revealed that imidacloprid treatment was no different from pyriproxyfen, and both were significantly inferior in efficacy than that observed for cyantraniliprole and flupyradifurone. The significant rate response of the imidacloprid bioassay indicated diminished mortality from the high concentration, suggesting a possible rise in resistance to this insecticide. If both the low and high concentrations control whiteflies, then the population is very susceptible to that active ingredient. Our field results of increased whitefly numbers in the imidacloprid-treated plots confirmed this.

In general, the insecticides used in this study provided a representative range for whitefly adult control efficacy from the most efficacious producing approximately 90% (laboratory) to 63% (field) control to the least efficacious resulting in less than a 45% (laboratory) to 25% (field) reduction in adults relative to the untreated check (Table 3 and Table 4). Based on whitefly survival in the bioassays, acetamiprid (4A) presented a shift between 2021 and 2022, indicating more pest susceptibility. As anticipated, the immature-targeting mode of action, pyriproxyfen (7C), was notably less effective against adults. In this study, survival rates of adults frequently approached 50% but never fell below 20% after 24 h exposure. Other insecticides with a neurotoxic mode of action, such as clothianidin (4A) and sulfoxaflor (4C), as well as flonicamid (29) and spiromesifen (23), presented moderate to low degrees of efficacy. In the 2021 field season, notably, squash plots had greater whitefly numbers than cucumber plots. In the 2022 season, cucumber plants experienced abundant numbers of adults, similar to squash.

## 4. Discussion

Our findings suggest that a low-cost and rapid bioassay has the potential to forecast insecticide efficacy against adult *Bemisia tabaci*. This has significant implications for decision making in chemical control, potentially saving growers a substantial amount of money. This whitefly species remains a pest of increasing importance in the world’s agricultural systems. In the state of Georgia, in the United States, cotton and vegetables are crops of major importance that are subject to infestations of this pest [15]. Cucurbits, including cucumber, squash, melons, and pumpkin, are among the main horticulture crops in Georgia; over 34,000 acres are cultivated with a production value of around 175 million USD in 2021 [16]. These crops are produced mainly in the southern region and are highly desirable hosts for *B. tabaci*. Moreover, *B. tabaci* serves as a vector for many viruses that cause diseases in these crops with significant economic damage [17,18]. Cucurbits are customarily cultivated in succession or adjacent to cotton during the warmest parts of the year, allowing a continuous migration of whiteflies between these different crop systems. This migration promotes consistent gene flow, particularly associated with resistant whitefly populations related to chemical control strategies for individual crops [19]. Thus, strategies for rapid efficacy surveys and resistance management of *B. tabaci* are needed.

Insecticides with fast-acting modes of action are highly desirable for many producers. In this experiment, the adoption of the adapted tube method proposed by [7] proved efficient for adult survival assessment due to its mobility and ease of setup, and it was easily tailored to our specific sampling needs. Thus, our methodology provides the option of rapid evaluation before treatment. As expected, the adult bioassays displayed different responses consistent with each respective insecticide mode of action. Similar results were found in *B. tabaci* adults treated with the maximum labeled rate for several insecticides [11]. The interval of 24 h after exposure provided a precise measure of the mortality response to be observed following a field application, and this response was insecticide specific. Therefore, an efficient laboratory determination of field responses to insecticide treatments is feasible for a given time interval. 

During this trial, we focused on ten insecticides from various IRAC groups (Table 1). Neurotoxic action insecticides (4A, 4C, 4D) generally produced higher and faster activity against adults under field and laboratory conditions. The rapid response occurs due to intrinsic characteristics of these products, such as broad spectrum, fast mode of action, and systemic action (plant tissue mobility), which makes them highly effective against whiteflies. As it imposes a high selection pressure on the pest population, factors such as these are essential to consider when implementing monitoring programs for whitefly control due to the risk of resistance build-up, with several reported cases so far [2,20,21]. Due to its haplodiploid characteristic, which confers complete dominance in resistant haploid males, *B. tabaci* populations develop resistance quickly, especially in enclosed environments or in isolated host systems where selection pressure is augmented, such as small vegetable production crop systems [22]. Therefore, quick assessment tools that approach the prediction of insecticide efficacy are increasingly needed in these cases.

Besides the neurotoxic products, cyantraniliprole, an anthranilic diamide, produced significantly reduced levels of whitefly adults but appeared not as fast-acting as the other insecticides. Following exposure, adults displayed sedentary behaviors lasting at least 24 h before “actual” death, confirmed upon lack of response after a light source was flashed for stimulation. The behavior of this insect in our study is aligned with previous reports that define the mode of action of the insecticide as a selective ryanodine receptor modulator, which ultimately acts over involuntary muscle contraction [1,23,24]. Therefore, the immobility observed was expected, but the time before mortality in *B. tabaci* was a newly reported observation. Interestingly, this means that in resistant populations, a cyantraniliprole response could lead to instances of observed knockdown without actual mortality.

Numbers of *B. tabaci* from field scouts in the summer and early fall of 2021 in Tift County, in southern Georgia, were lower than in previous years. In 2021, there was higher average rainfall than the previous year [25], which might have contributed to this decline in numbers. Moreover, the 2022 season was less severe in terms of rainfall, and whitefly numbers were significantly higher, suggesting a different set of climatic factors that might have affected field populations. Consistent with the laboratory results, the field scout data also reflected a change in efficacy response to imidacloprid, suggesting a possible rise in resistance to this insecticide, and also flupyradifurone, cyantraniliprole, and dinotefuran relative to each other. We observed a higher preference for squash based on *B. tabaci* field numbers in that season. In the 2022 season, however, whitefly numbers were more consistent between host plants, and there was a more rapid re-invasion.

Laboratory bioassay results proved to be good indicators for evaluating whitefly population response to insecticides, potentially improving decision making for vegetable growers attempting to manage resistance with effective insecticide rotations. For future studies, bioassay improvement, possibly using a critical low concentration to anticipate the rise in tolerance to a particular mode of action, should be pursued. We propose that a rate response between the high and low insecticide concentration indicates potential weakness in a product’s efficacy in the field and should be considered as a clarifying addendum to the maximum dose bioassay method. Additionally, the technique should be tested with different whitefly populations to confirm if it helps pest managers improve whitefly control and reduce the carryover of resistance genes. In summary, maximum dose-based bioassays as rapid test techniques are potentially a more efficient way to investigate insecticide resistance spread than generating LC_50_ curves for populations of adults from each field.

## 5. Conclusions

Determining the efficacy of field insecticides on *B. tabaci* is a critical step towards controlling this pest and minimizing the damage it can cause to crops worldwide. Maximum dose bioassays are a useful tool for measuring the effectiveness of insecticides in field conditions and can provide valuable insights into the optimal dosages required for effective control. By conducting relatively quick and easy-to-set bioassays, farmers and researchers can gain insight into the potency and effectiveness of different insecticides on a given whitefly population, taking account of the dynamism of its genetic characteristic prone to insecticide resistance. This paper highlights the importance of using maximum dose bioassays in the field to accurately determine insecticide efficacy and optimize control strategies for whiteflies.

## Figures and Tables

**Table 1:** Description of insecticide treatments based on commercial labeled rate and overall ranking for the control of whitefly adults in field and laboratory experiments in Georgia, 2021–2022.

IRAC ^1^ Group	Common Name	Commercial™ Name	Per Hectare Rate	Bioassay Rate (p.p.m. of a.i. ^2^)
-	Water check	-	-	-
4A	Imidacloprid	Admire Pro 4.6F	160.8 mL	73.6
4A	Dinotefuran	Venom 70SG	280.2 g	209.4
4A	Acetamiprid	Assail 30SG	280.2 g	89.7
4A	Clothianidin	Belay 50WDG	292.3 mL	156.8
4C	Sulfoxaflor	Transform WG	157.6 g	84.1
4D	Flupyradifurone	Sivanto Prime 1.67SL	876.9 mL	160.3
7C	Pyriproxyfen	Knack 0.86EC	730.8 mL	87.7
29	Flonicamid	Beleaf 50SG	299.8 g	160.3
23	Spiromesifen	Oberon 2SC	621.1 mL	153.4
28	Cyantraniliprole	Exirel 0.83SC	986.5 mL	107.6

^1^ Insecticide Resistance Action Committee. ^2^ Parts per million of active ingredient.

**Table 2:** Treatment and replication effects based on analysis of variance for surviving adult *Bemisia tabaci* on cucurbit crops in a field experiment at the Coastal Plain Experiment Station, Tifton, GA.

Crop System		24 h Pre-Treatment Bioassay		24 h Post-Treatment Field	
		F	*p* > F	F	*p* > F
Squash #1 2021					
	Insecticide	23.4	<0.001	16.5	<0.001
	Rep/block	2.17	0.072	0.87	0.484
Squash #2 2021					
	Insecticide	16.4	<0.001	7.81	<0.001
	Rep/block	1.71	0.150	4.16	0.003
Cucumber #1 2021					
	Insecticide	19.7	<0.001	13.5	<0.001
	Rep/block	3.00	0.020	1.52	0.199
Cucumber #2 2021					
	Insecticide	55.3	<0.001	6.5	<0.001
	Rep/block	3.10	0.026	0.31	0.873
Squash #3 2022					
	Insecticide	9.6	<0.001	10.8	<0.001
	Rep/block	3.36	0.011	0.41	0.803
Squash #4 2022					
	Insecticide	9.7	<0.001	7.3	<0.001
	Rep/block	1.27	0.293	0.91	0.458
Cucumber #3 2022					
	Insecticide	11.9	<0.001	2.7	0.005
	Rep/block	1.85	0.121	0.35	0.846
Cucumber #4 2022					
	Insecticide	10.5	<0.001	8.39	<0.001
	Rep/block	0.32	0.898	2.01	0.096

Note: Df values of insecticide and replicate, respectively, for Cucumber #1 = 10 and 4, all others = 10 and 5.

**Table 3:** Mean laboratory and field proportional survival (% ± SE) of *Bemisia tabaci* populations in squash experiments and associated bioassays under controlled conditions (25 ± 2 °C, 60 ± 5% RH, 24 h L:D), Tifton, GA, 2021–2022.

Treatment	24 h Laboratory Bioassay (%)		24 h Field Trial (%)	
2021	Squash #1	Squash #2	Squash #1	Squash #2
Check	89.32 ± 2.60 a ^1^	87.79 ± 7.41 a	42.76 ± 4.53 a	37.39 ± 4.84 a
Pyriproxyfen	56.64 ± 8.05 b	54.58 ± 10.19 b	18.41 ± 2.40 bcd	28.84 ± 5.27 ab
Clothianidin	32.96 ± 4.35 bcd	43.93 ± 4.01 bc	24.53 ± 3.86 b	39.13 ± 4.71 a
Spiromesifen	39.45 ± 9.49 bc	29.58 ± 8.24 bcd	21.35 ± 3.42 bc	30.65 ± 6.73 ab
Flonicamid	19.10 ± 6.90 cde	26.33 ± 4.67 bcd	26.65 ± 2.35 b	18.90 ± 2.53 bc
Acetamiprid	15.51 ± 4.10 cde	24.20 ± 5.81 cd	11.71 ± 1.02 cde	14.35 ± 4.65 bc
Sulfoxaflor	17.29 ± 3.50 cde	22.14 ± 9.82 cd	19.06 ± 1.67 bcd	30.32 ± 4.63 ab
Imidacloprid	16.29 ± 4.84 cde	11.42 ± 3.57 d	20.88 ± 2.13 bc	21.68 ± 2.82 abc
Flupyradifurone	11.40 ± 5.56 de	8.59 ± 4.19 d	5.41 ± 0.58 e	8.45 ± 1.47 c
Cyantraniliprole	7.39 ± 3.52 e	9.52 ± 4.21 d	12.53 ± 0.97 cde	18.65 ± 1.86 bc
Dinotefuran	7.83 ± 2.28 e	2.65 ± 1.42 d	9.29 ± 0.90 ed	6.10 ± 1.01 c
2022	Squash #3	Squash #4	Squash #3	Squash #4
Check	84.54 ± 4.37 a	76.45 ± 6.34 a	66.22 ± 4.25 a	51.76 ± 4.64 a
Pyriproxyfen	50.06 ± 8.71 b	31.73 ± 6.61 bcd	51.33 ± 3.60 abc	41.91 ± 4.16 ab
Clothianidin	36.23 ± 8.40 bc	54.73 ± 11.40 ab	62.11 ± 4.65 ab	51.03 ± 5.71 a
Spiromesifen	49.73 ± 12.97 b	50.89 ± 9.79 abc	51.22 ± 4.50 abc	36.32 ± 4.87 abc
Flonicamid	40.20 ± 8.80 bc	55.24 ± 9.76 ab	44.44 ± 4.04 bcd	36.03 ± 4.45 abc
Acetamiprid	23.03 ± 1.79 bc	11.82 ± 3.06 d	49.56 ± 3.89 abc	28.53 ± 2.59 bc
Sulfoxaflor	32.18 ± 5.79 bc	20.42 ± 4.57 cd	52.00 ± 4.75 abc	49.85 ± 4.95 a
Imidacloprid	33.36 ± 7.06 bc	29.16 ± 6.49 bcd	39.00 ± 3.26 cd	49.71 ± 4.14 a
Flupyradifurone	20.08 ± 2.99 bc	10.95 ± 3.54 d	28.44 ± 2.25 d	22.35 ± 2.09 c
Cyantraniliprole	15.25 ± 4.11 c	20.34 ± 5.62 cd	30.67 ± 3.81 d	28.09 ± 3.11 bc
Dinotefuran	18.85 ± 4.83 c	14.79 ± 5.49 d	30.56 ± 2.28 d	26.76 ± 2.31 bc

^1^ Means followed by the same letter within each column are not significantly different (*p* > 0.05, Tukey’s test).

**Table 4:** Mean laboratory and field proportional survival (% ± SE) of *Bemisia tabaci* populations in cucumber experiments and associated bioassays under controlled conditions (25 ± 2 °C, 60 ± 5% RH, 24 h L:D), Tifton, GA, 2021–2022.

Treatment	24 h Laboratory Bioassay (%)		24 h Field Trial (%)	
2021	Cucumber #1	Cucumber #2	Cucumber #1	Cucumber #2
Check	75.45 ± 8.81 a ^1^	68.66 ± 2.81 a	60.44 ± 3.73 a	50.97 ± 4.43 a
Pyriproxyfen	21.69 ± 4.26 b	49.67 ± 3.75 b	61.76 ± 3.74 a	41.61 ± 4.37 abc
Clothianidin	24.47 ± 4.59 b	41.20 ± 6.97 b	39.71 ± 3.86 bc	44.84 ± 3.98 ab
Spiromesifen	21.03 ± 3.47 b	16.45 ± 4.87 c	34.85 ± 3.32 bcd	40.81 ± 4.01 abcd
Flonicamid	18.55 ± 5.13 b	22.27 ± 4.96 bc	45.29 ± 4.21 ab	40.81 ± 4.61 abcd
Acetamiprid	14.26 ± 3.58 b	11.23 ± 0.80 d	35.15 ± 4.35 bcd	45.32 ± 3.76 ab
Sulfoxaflor	12.68 ± 3.90 b	19.37 ± 5.10 c	43.24 ± 4.76 b	40.32 ± 3.73 abcd
Imidacloprid	15.44 ± 4.08 b	4.04 ± 1.19 d	32.79 ± 2.91 bcd	32.42 ± 3.91 bcde
Flupyradifurone	11.67 ± 1.83 b	1.93 ± 2.22 d	31.91 ± 3.08 bcd	24.03 ± 2.23 de
Cyantraniliprole	9.66 ± 2.45 b	4.56 ± 2.22 d	24.26 ± 2.99 cd	21.77 ± 2.16 e
Dinotefuran	16.09 ± 3.47 b	1.95 ± 1.66 d	19.12 ± 2.04 d	26.45 ± 2.66 cde
2022	Cucumber #3	Cucumber #4	Cucumber #3	Cucumber #4
Check	83.09 ± 5.37 a	73.07 ± 8.24 a	31.20 ± 2.70 a	52.63 ± 3.07 a
Pyriproxyfen	42.16 ± 9.08 bc	50.12 ± 7.89 ab	25.06 ± 3.21 ab	41.88 ± 3.21 abc
Clothianidin	46.94 ± 8.14 b	56.83 ± 11.72 ab	30.76 ± 4.24 ab	49.25 ± 2.89 a
Spiromesifen	30.40 ± 8.37 bcde	58.60 ± 7.64 a	28.29 ± 3.23 ab	50.50 ± 3.73 a
Flonicamid	34.89 ± 6.17 bcd	43.28 ± 12.99 abc	26.58 ± 3.15 ab	49.88 ± 4.05 a
Acetamiprid	26.52 ± 5.70 bcde	20.98 ± 4.70 bcd	24.30 ± 2.28 ab	32.75 ± 2.90 bcd
Sulfoxaflor	34.16 ± 9.67 bcde	12.76 ± 3.51 cd	23.73 ± 3.93 ab	43.13 ± 3.91 ab
Imidacloprid	23.01 ± 5.58 bcde	40.45 ± 7.06 abcd	28.10 ± 5.80 ab	46.50 ± 3.18 ab
Flupyradifurone	4.81 ± 1.60 e	6.50 ± 2.35 d	15.76 ± 1.90 ab	27.88 ± 2.25 cd
Cyantraniliprole	15.82 ± 3.45 cde	5.08 ± 1.82 d	15.57 ± 2.02 b	38.38 ± 3.49 abcd
Dinotefuran	8.05 ± 2.17 de	6.66 ± 1.84 d	19.43 ± 2.07 ab	26.63 ± 2.22 d

^1^ Means followed by the same letter within each column are not significantly different (*p* > 0.05, Tukey’s test).

## Data Availability

The data presented in this study are available on the request of the corresponding authors.
